# Effectiveness of Biologically Inspired Neural Network Models in Learning and Patterns Memorization

**DOI:** 10.3390/e24050682

**Published:** 2022-05-12

**Authors:** Lorenzo Squadrani, Nico Curti, Enrico Giampieri, Daniel Remondini, Brian Blais, Gastone Castellani

**Affiliations:** 1 Department of Physics and Astronomy, University of Bologna, 40126 Bologna, Italy; lorenzo.squadrani@studio.unibo.it (L.S.); daniel.remondini@unibo.it (D.R.); 2 Department of Experimental, Diagnostic and Specialty Medicine, University of Bologna, 40126 Bologna, Italy; nico.curti2@unibo.it (N.C.); gastone.castellani@unibo.it (G.C.); 3 INFN, 40127 Bologna, Italy; 4 Department of Science, Bryant University, Smithfield, RI 02917, USA; bblais@bryant.edu

**Keywords:** machine learning, neural networks, optimization, entropy, learning algorithm

## Abstract

**Purpose:** In this work, we propose an implementation of the Bienenstock–Cooper–Munro (BCM) model, obtained by a combination of the classical framework and modern deep learning methodologies. The BCM model remains one of the most promising approaches to modeling the synaptic plasticity of neurons, but its application has remained mainly confined to neuroscience simulations and few applications in data science. **Methods:** To improve the convergence efficiency of the BCM model, we combine the original plasticity rule with the optimization tools of modern deep learning. By numerical simulation on standard benchmark datasets, we prove the efficiency of the BCM model in learning, memorization capacity, and feature extraction. **Results:** In all the numerical simulations, the visualization of neuronal synaptic weights confirms the memorization of human-interpretable subsets of patterns. We numerically prove that the selectivity obtained by BCM neurons is indicative of an internal feature extraction procedure, useful for patterns clustering and classification. The introduction of competitiveness between neurons in the same BCM network allows the network to modulate the memorization capacity of the model and the consequent model selectivity. **Conclusions:** The proposed improvements make the BCM model a suitable alternative to standard machine learning techniques for both feature selection and classification tasks.

## 1. Introduction

There are increasing applications of neural network models in data science analysis, due to their capability of easily establishing nonlinear correlations between data. The emerging problem of models explainability is a direct consequence of the trend which leads modern artificial intelligence research and of the demand for even more performing models, neglecting a complete understanding of what happens inside them. This trend partially contrasts the original idea behind neural network models, which aimed to mathematically formalize the biology behind neuronal cells. Starting from 2020, the European Commission (EC) published the Checklist for Trustworthy Artificial Intelligence [1], in which it established as primary requirements for artificial intelligence applications in medical research the traceability and explainability of the artificial intelligence models. Therefore, model explainability is becoming even more important in research applications which require a precise response to the decisions made by the model [2].

Back-propagation algorithm (BPa) is the standard method used to estimate the error-driving updates of the model parameters. Due to its simplicity and computational efficiency, BPa remains the standard method for the evaluation of neural network updates. BPa can manage neural network models with arbitrary depth, encouraging machine learning developers to build even more complex architectures, until their required performances are achieved.

In contrast with the large diffusion of models trained with BPa, there is a second class of architectures which base their efficiency on the enrichment of the requirements of the learning procedure, which goes further with respect to a simple optimization loss function, but, instead, includes additional desired properties. We can roughly divide these models into two subclasses: physically/mathematically and biologically inspired models. Belonging to the first class, we have architectures such as Boltzmann machine [3] and Belief Propagation models [4,5], which base their functionalities on properties related to statistical distributions and physics behaviors (e.g., Ising model, magnetization, and spin-glasses). The interpretability of the learning process of these architectures relies on a strict mathematical formalism for the description of physical properties and on the formalization of concepts such as memory and statistical equilibrium. In the second class, we find the historical starting points of neural network applications, such as the Hodgkin–Huxley model [6], Rosenblatt’s perceptron [7], until the modern neuroscience network architectures [8,9,10,11]. The merit of all these models lies in their exceptional adherence to the biology (electro-physiological and molecular) experiments on neuronal cells. Their aim is, in fact, to mimic, as much as possible, the functionalities of neuronal cells and their interaction with the human brain. The ability to work with biologically inspired models allows a continuous integration between laboratory results and computational simulations in discovering novel functionalities of the human brain.

Almost all biologically inspired models are based on the concept of synaptic plasticity, i.e., the activity-based modification (potentiation/depression) of synaptic connections between neurons. This mechanism represents one of the most important properties of neuron cells since it is at the base of their learning and memorization capability [12]. Synaptic plasticity was theoretically conjectured and modeled by Hebb in 1949. The Hebb model has found experimental agreement in several neuroscience experiments, successfully reproducing the development of neuron selectivity. A remarkable contribution to this topic was made by E. Bienenstock, L. Cooper, and P. Munro, who introduced a biologically inspired neuron model, i.e., the BCM model, able to describe multiple synaptic features of the cortical neurons. The BCM model formulation started from Hebb’s rule for the description of memory formation and computational adaption of brain neurons, and it describes the evolution of neurons states via a set of time-dependent evolution. The BCM model remains one of the most promising approaches to modeling the synaptic plasticity of neurons and, from its first formulation, several improvements have been proposed to address computational and stability problems [13,14]. Each improvement of the model has always been evaluated by balancing the model performances with a corresponding biological interpretation of the neurons’ behavior, verifying the predictions by neuroscience experiments [15].

The dynamic characteristics of the BCM model have been studied extensively by several authors [16,17], aiming to understand and explain neuron functionalities and improve the memorization capability. The analogy with the well-known dimensionality reduction techniques was mathematically proved only for a small set of neurons and the results confirmed by numerical simulations [18]. Despite its computational efficiency, the application of the BCM model has been limited to theoretical neurobiological studies, with few applications in data science. Starting from its formulation, the BCM model found applications as a computational framework developed to prove *ad hoc* theories or validate experiments. Its application to non-experimental data, i.e., deriving from different applications or research fields, is still limited.

The work of Krotov et al. [19] was one of the first proposals of its application on real data. Krotov et al. introduced a modified version of the BCM model, designed to improve numerical performance, proving the efficiency of the synergy between biologically inspired and standard BPa neural networks in classification tasks.

Krotov et al. introduced a learning algorithm (*KH* in the following) that uses three ideas: a BCM-like learning rule, competition between the hidden units, and a homeostatic constraint on the synaptic weights. There is competition between the *K* hidden neurons to choose which neuron to alter its synapses. At each new stimulus step, there is competition between neurons to determine which will “win” and have its synapses changed. *KH* is intrinsically a *K*-body problem, not a one-body problem like BCM. Unlike BCM, which features competition between the input patterns, *KH* additionally involves competition between the hidden neurons.

In this work, we propose an implementation of the BCM model, obtained by the combination of the classical framework and deep learning features. Our aim is to provide an integration of the results obtained by deep learning research into the BCM model. We started from the original implementation of the BCM model, and we extended it, studying its performance in relation to different activation functions, weights initialization, and optimization algorithms [20]. The biological analogy with the neurons’ behavior is preserved, keeping fixed the core functionalities of the model, but a better and faster convergence is proved. The proposed improvements allow us to apply the BCM model to real datasets, making it a suitable alternative to standard machine learning techniques.

## 2. Materials and Methods

### 2.1. Mathematical Framework

The BCM model describes the synaptic plasticity via a dynamic adaptation depending on the post-synaptic activity. The behavior of cortical neurons is explained by a combination of long-term potentiation and long-term depression given by a series of stimuli applied to presynaptic neurons [21]. Starting from the Hebbian learning rule, which establishes that repeated and persistent activities could determine a transmission of information between neurons, the BCM model aims to overcome mathematical issues related to the stability and applicability of neuron models.

In this work, we refer to the BCM implementation proposed by Law and Cooper in 1994 [14], which is described by the set of equations

(1) $$\begin{array}{cc} z & =\sigma \left(\sum_{i}w_{i}x_{i}\right)\\ \theta & =E[z^{2}]\\ \frac{dw_{i}}{dt} & =\frac{z\left(z-\theta \right)x_{i}}{\theta } \end{array}$$

where $z_{i}$ and σ are the postsynaptic activity of the *i*-th neuron and a nonlinear activation function, respectively. The value of θ, commonly referred to as *modification threshold*, represents a long-term average of the synaptic activation.

Shouval et al. [22] proved the high selectivity of artificial neurons trained by BCM equations: synaptic connections tend to produce highly oriented receptive fields during the training, making neurons responsive to only a subset of the provided patterns. Several authors extended these results also to network architectures of BCM neurons [23,24], highlighting the presence of receptive fields in neurons synapses.

**Figure 1:**
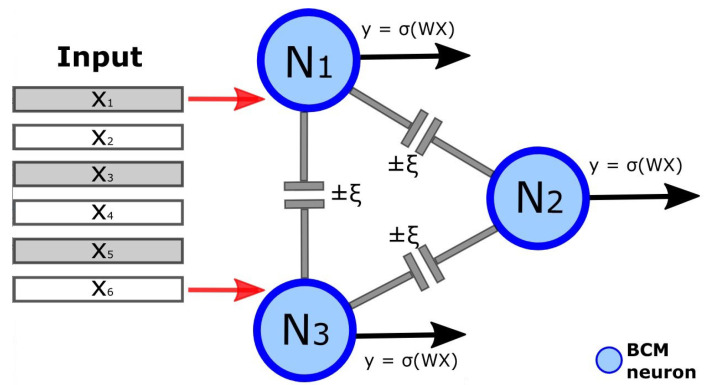
Schematic representation of a BCM network composed of 3 neurons. Cortico-cortical connections are represented with gray lines between neurons, with ±ξ strength. Presynaptic activities (input) and postsynaptic activities (output) are represented by red and black arrows, respectively. Postsynaptic activities are computed according to the BCM equations.

Castellani et al. [16] studied the classical BCM model including lateral connections and nonlinearity between neurons. Lateral connections would allow to inhibit/increment the postsynaptic activities in relation to the state of neurons neighborhood, including competition and cooperation between neurons. In other words, it involves the introduction of an extra matrix term (L), which influences the postsynaptic vector as

(2) $$z=\sigma \left({\left(1-L\right)}^{-1}WX\right)$$

where *W* and *X* are the synaptic weights matrix and the input matrix, respectively. The term L represents the cortico-cortical connectivity matrix in which $l_{ij}$ are the interaction strength factors (±ξ) between each pair of neurons. Keeping fixed the interaction strength between neurons, the L matrix is built setting all the elements equal to ξ and 0 on the diagonal (avoiding self-interactions). Positive weights of the L matrix correspond to cooperation between neurons, while negative weights indicate competition. The choice of setting all lateral connections to the same value was made to allow for a more interpretable set of results from this study. The exploration of some of the potentially infinite distribution of synaptic weights was deemed to be outside the scope of this work. A schematic representation of a simple 3-neuron BCM network is shown in Figure 1.

The introduction of lateral connections determines the level of competitiveness between BCM neurons. Inhibitory lateral connections would tend to discourage neurons from memorizing the same patterns, while positive lateral connections increase the probability of several neurons reaching the same stationary state. Therefore, the strength of lateral interaction directly determines the learning capacity of the model.

#### 2.1.1. Optimization Strategy

The BCM model does not fix any constraints on the optimization strategy to choose for the synaptic convergence. In order to improve the convergence efficiency, we combine the plasticity rule with the optimization tools of modern deep learning models. In particular, we apply the Adam optimization algorithm [25], using random batch subdivisions of the training patterns and performing the update of model parameters at every batch.

The mathematical framework of the BCM model establishes that postsynaptic activity is given by a linear combination of synaptic weights and inputs, processed by an activation function. No constraints on the form of the activation function are posed: to achieve nontrivial results, the nonlinearity could be imposed, while for the biological interpretation, the positivity is required. Historically, the classical formulation of the model uses a logistic activation function, following the trend proposed by other neuroscience applications. The effect of activation function on performances and learning of deep neural network models has been discussed by several authors [26,27,28]. Different mathematical equations have been proposed to address numerical and stability issues related to the training of complex models [29,30,31]. Currently, the most promising results have been obtained by the ReLU (*Rectify Linear Unit*) activation function [32]. Its usage is attributed to its numerical efficiency and to the benefits it brings, in terms of information disentangling, information representation, sparsity, and reduction of vanish gradient effect [33]. According to the previous considerations, we activate BCM neurons using ReLU activation function.

#### 2.1.2. Modification Threshold

The modification threshold θ of neurons is one of the key aspects of the BCM algorithm. The choice of how to compute it determines whether neurons converge to a nontrivial stationary state and the properties of such states. In our implementation, we use a moving average of previous batch-averaged quadratic postsynaptic activities, i.e.,

(3) $$\theta_{t}=\gamma \theta_{t-1}+\left(1-\gamma \right){\langle z^{2}\rangle }_{b_{t}}$$

where γ is the decay-memory factor, and ${\langle \cdot \rangle }_{b_{t}}$ is the average over the batch of training patterns considered at the time step *t*. The superlinearity with respect to *z* ensures the convergence of the neuron [34]. The final selectivity reached by the neuron depends on the choice of the memory factor value. In particular, values close to 1 enforce the neuron to develop high selectivity, while smaller values lead to lower selectivity. The same behavior is obtained by setting γ=0 and varying the training batch size. This is due to the equivalence between the average over a sufficiently large time window and the average over a sufficiently large portion of the “environment” (training set).

The threshold as a function of time $\theta_{t}$ is used to monitor the convergence of each neuron. The resulting trend could be very noisy according to the choice of the batch size and the memory factor. To obtain smoother and easy-to-read curves, we look at the average of θ over each epoch, i.e.,

(4) $$\langle \theta \rangle =\frac{1}{B}\sum_{t=1}^{B}\theta_{t}$$

where *B* is the number of steps in one epoch.

### 2.2. Network Properties

Given a set of *N* interconnected neurons, i.e., a BCM network, the learning power of the architecture is determined by two fundamental properties: the neurons’ *selectivity* and the neurons’ *competitiveness*.

#### 2.2.1. Selectivity

We say that the *i*th neuron has selected the pattern x∈X if

(5) $$z_{i}\left(x\right)-E\left[z_{i}\left(x\right)\right]>0$$

where $z_{i}\left(x\right)$ is the postsynaptic activity of the *i*th neuron to the *x* pattern, and $E[z_{i}]$ is the expectation value over *X*. We measure the neuron selectivity $\alpha_{i}$ by counting the number of training patterns “selected” by the neuron, hence

(6) $$\alpha_{i}=\left|A_{i}\right|,\;A_{i}=\left\{x\in X|z_{i}\left(x\right)-E\left[z_{i}\left(x\right)\right]>0\right\}$$

The selectivity is a property of a single neuron, and neurons in the same BCM network can develop different selectivity levels.

#### 2.2.2. Competitiveness

We evaluate the level of competitiveness of a BCM network by measuring the overlap among the set of patterns selected by the neurons. Let *S* be the total number of patterns which have been selected by the BCM network, and $\bar{\alpha }$ the average selectivity of the neurons in the network. We define the *overlapping index* as

(7) $$\beta =\frac{S}{\bar{\alpha }}$$

where β ranges in [1,N]. The complete absence of overlap, which coincides with the maximum level of competitiveness, is equal to the number of neurons considered.

#### 2.2.3. Memorization

For a fixed number of neurons and their selectivity $\alpha_{i}$, the maximum memorization capacity of the model, i.e., the maximum number of patterns that can be selected, is given by

(8) $$C=\sum_{i=1}^{N}\alpha_{i}\approx \bar{\alpha }\times N$$

The effective number of patterns that are selected, i.e., the effective memorization capacity, is determined by the competitiveness β of the network, measured through the overlapping index.

Considering a set of *T* different patterns, the following cases can occur:

- T<<C. In this configuration, the model can reach a perfect memorization of all the patterns, with redundancy. The influence of lateral connections could be negligible.
- T≈C. In this configuration, the model can reach a perfect memorization of all the patterns, without redundancy. The importance of the lateral connections becomes crucial for the memorization, since the probability of having multiple neurons at the same stable point is not negligible and having them would imply the loss of some patterns.
- T>>C. The model cannot reach a perfect memorization of the patterns since the problem is ill-posed, by definition. There is a probability of obtaining two (or more) neurons at the same stable points proportional to the strength of lateral connections imposed.

## 3. Results

We apply the optimized version of the BCM model with 100 neurons on MNIST and CIFAR-10 datasets. Both datasets are standard toy models for the benchmarking of machine learning performances. Furthermore, the same datasets were also used by Krotov et al. [19] for the validation of their modified version of the BCM algorithm.

We performed the simulations according to the optimization strategy proposed in the above sections, evaluating the model convergence using Equation (4) and testing the learning capacity of the model using Equation (8). Details on data preprocessing and model hyperparameters used for the model training can be found in the Appendix A. All the results are reproducible using the developed code, publicly available on Github [20], where more detailed images and animations of the results can be seen.

### 3.1. MNIST Dataset

The dataset includes 50,000 grayscale images (28×28) of handwritten digits. The dataset contains 10 classes of images, according to the ten putative digits ∈[0,9]. For each class, an equal number of samples is provided, ensuring a reasonably good balancing between the classes.

#### 3.1.1. Synaptic Weights

**Figure 2:**
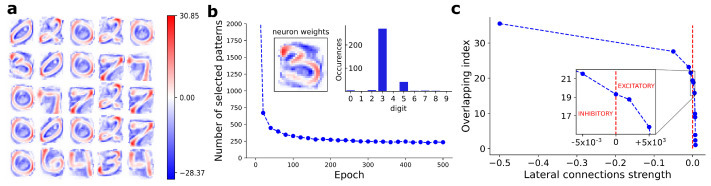
(**a**) Weights learned by 25 arbitrarily chosen BCM neurons after convergence on the MNIST dataset. (**b**) Number of training patterns selected according to the definition proposed in Equation (5), as a function of training epochs, for an arbitrarily chosen BCM neuron. Top right: number of patterns selected by the considered neuron, split according to the ten putative digits included in the MNIST dataset. Top left: weights bitmap of the considered BCM neuron and corresponding to the most selected digit. (**c**) Observed overlapping index (β, Equation (7)) as a function of lateral connections strength. As expected, β score progressively grows with a monotonic trend as the inhibitory strength of lateral connections is increased. β score rapidly reaches the maximum overlap value, equal to 1, with just small values of excitatory lateral connections.

We show in Figure 2a the bitmaps of weights learned by 25 arbitrarily chosen BCM neurons, at the end of 500 training epochs. We checked the convergence of BCM neurons using Equation (4).

#### 3.1.2. Selectivity

We evaluated the neurons’ selectivity considering a set of independent neurons, i.e., turning off the lateral connections. For each BCM neuron, we estimated the selectivity level according to Equation (5). In Figure 2b, we show the number of selected patterns as a function of the training epochs, for an arbitrarily chosen neuron. On the top right of Figure 2b, we show the number of patterns selected by that neuron, split according to the ten putative digits. The majority of selected patterns represents the same handwritten digit, visible also in the corresponding weights bitmap (see top left of Figure 2b).

#### 3.1.3. Competitiveness

In Figure 2c, we show the β score (Equation (7)) as a function of the lateral connection strengths imposed between BCM neurons. As inhibitory lateral connections increase, the β score progressively grows with a monotonic trend, leading to a reduction of the overlap. When excitatory (positive) lateral connections are used, the β score rapidly converges to 1, i.e., the maximum overlap.

### 3.2. CIFAR-10 Dataset

The dataset includes 70,000 general purpose RGB images (32×32). The dataset contains 10 classes of images, including natural images. For each class, an equal number of samples is provided, ensuring a reasonably good balancing between the classes.

#### 3.2.1. Synaptic Weights

**Figure 3:**
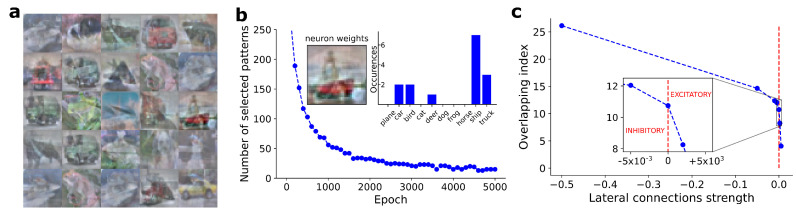
(**a**) Weights learned by 25 BCM neurons after convergence on CIFAR-10 dataset. (**b**) Number of training patterns selected according to the definition proposed in Equation (5), as a function of training epochs, for an arbitrarily chosen BCM neuron. Top right: number of patterns selected by the considered neuron, split according to the ten putative classes included into the CIFAR-10 dataset. Top left: weights bitmap of the considered BCM neuron, and corresponding to the selected class. (**c**) Observed overlapping index (β, Equation (7)), as a function of lateral connections strength. As expected, β score progressively grows with a monotonic trend as inhibitory strength of lateral connections are increased. β score rapidly reaches the maximum overlap value, equal to 1, with just small values of excitatory lateral connections.

In Figure 3a, we show the synaptic weights learned by 25 BCM neurons, at the end of 5000 training epochs. We checked the convergence of BCM neurons using Equation (4). The weights visualization was obtained by scaling the synaptic values into the range [0,255].

#### 3.2.2. Selectivity

We evaluated the neurons’ selectivity considering a set of independent neurons, i.e., turning off the lateral connections. For each BCM neuron, we estimated the selectivity level according to Equation (6). In Figure 3b, we show the number of selected patterns as a function of the training epochs, for an arbitrarily chosen neuron. On the top right of Figure 3b, we show the number of patterns selected by the neuron, split according to the ten putative classes. The maximum responsive class corresponds to the one visible in the weights bitmap (see top left of Figure 3b).

#### 3.2.3. Competitiveness

In Figure 3c, we show the trend of β score (Equation (7)) in relation to the lateral connection strengths imposed between BCM neurons. The β score monotonically grows according to inhibitory lateral connections, following a trend equivalent to the one obtained in the MNIST dataset.

## 4. Discussion and Conclusions

In this work, we proposed an optimized and reviewed version of the BCM model, obtained by the integration of modern deep learning features into its classical framework. Until now, the applications of the BCM model have mainly involved neuroscience simulations as a benchmark of biological theories, with few applications to data science. Starting from the work of Krotov et al. [19], the application of biologically inspired models as machine learning alternatives is acquiring increasing interest. The *explainability* requirement, even more imposed in deep learning applications, is leading to a rediscovery of easy-to-understand models in managing machine learning tasks. In this work, we numerically proved that BCM neurons satisfy significant requirements on this topic.

The optimizations introduced in this work have improved the model training efficiency. In our simulations (excluded from this work for sake of brevity), we tested several kinds of activation functions (e.g., SeLU, Loggy, Tanh, etc.), but they were all outperformed by the ReLU one. In detail, we observed a significant increment in neuron selectivity by the introduction of ReLU activation function, allowing the network to achieve better stable states than the classical logistic activation function. Furthermore, the use of Adam optimization algorithm, for the update of synaptic weights, produces faster convergence compared to the standard Stochastic Gradient Descent algorithm, without affecting neuron selectivity. For both numerical simulations on MNIST and CIFAR-10 datasets, the visualization of neuron synaptic weights has confirmed the memorization of a subset of patterns.

We proved a high level of selectivity by BCM neurons, for both MNIST and CIFAR-10 datasets. The selectivity obtained by BCM neurons must not be associated to a simple memorization of the patterns. Looking at the neurons’ synaptic weights, the stored patterns are partially distorted, despite being clearly recognizable for human eyes. In our simulations (excluded from this work for sake of brevity), we tested the perfect memorization of the neurons, manually setting the synaptic weights equal to a subset of training patterns. The evaluation of the introduced selectivity score in this extreme case was far lower than the obtained-by-training one. This behavior confirms that BCM neurons do not simply “memorize” the provided patterns, but they perform an internal feature extraction procedure for pattern clustering.

The patterns encoded in the synaptic weights store features of input images shared by a putative group of patterns. The level/order of features encoded is directly determined by the level of selectivity obtained by the neuron at convergence. We remark that the level of neuron selectivity is related to the complexity of the training set and the number of neurons used. Neuron selectivity can be tuned according to the introduced *memory factor*: for the sake of brevity, we did not show the results obtained on these trends, but they can be easily reproduced using the developed code.

There is evidence that neurons exhibit different levels of selectivity [35] in biological systems. We found an equivalent differentiation in the BCM framework considering the neurons’ memory capacity. This behavior confirms the biological validity of the BCM model, which is preserved by the introduction of the proposed improvements.

The results proposed in Figure 2b show the evidence of a pattern-like image stored into synaptic weights of BCM neuron. We estimated 250 patterns at which the considered neuron responds, of which 201 are labeled as digit-3. The remaining patterns are false positive classifications in terms of digit recognition. However, looking at the bitmap of neuron synaptic weights, we clearly see a uniformity in the digit shape, with high-intensity areas (red spots) located in well determined positions. These areas represent the keypoints learned by the neuron for the discrimination between the subset of selected patterns and the remaining ones.

Analogous results have been found on the CIFAR-10 dataset (see Figure 3b). In this case, neuron synaptic weights are less clear-cut, but still recognizable by humans. In the same way, the number of patterns selected by the neuron is also smaller, confirming the greater complexity of the CIFAR-10 dataset compared to the MNIST one. In our simulations (excluded from this work for the sake of brevity), we could obtain a level of neuron selectivity compatible with the MNIST dataset also for the CIFAR-10 case, but with a significant loss in the human interpretability of the weights bitmap. The large number of degrees of freedom and the variability of CIFAR-10 patterns impose a harder feature extraction task, which is reflected in the not-perfect patterns memorized by neurons. The CIFAR-10 dataset is commonly used for the training of object detection models, and it represents an intermediate benchmark between the (simpler) MNIST dataset and real data applications. The CIFAR-10 dataset involves more complex patterns and textures than the MNIST ones, with an order of magnitude more data to process. The correlation between the three image channels, due to pixel colors, implies long-range correlation between the model weights, requiring a deeper learning capability by the model. The use of benchmark datasets allows to show and understand exactly what BCM neurons have learned during the training procedure. This behavior is characteristic of biologically inspired models, while it is partially lost in modern deep learning applications [19]. A human-interpretable visualization of synaptic weights improves the possible explanation of the decisions made by automated systems and it provides an interface in developing improvements for model training and efficiency. We remark that feature extraction is performed by the BCM network in completely unsupervised training, leaving to the model the possibility or ability of finding features related to groups of patterns belonging to the same class.

While the selectivity and the number of neurons completely determine the maximum memorization capacity of the model (Equation (8)), the effective capacity depends on the level of competitiveness between neurons. The overlapping index reported in Figure 2 and Figure 3c shows that competitiveness can be implemented through lateral connections and it is proportional to their strength ξ. In particular, the memorization capacity nearly double, growing from about 19% to over 35% of the patterns for MNIST, and from 12% to 26% for CIFAR-10. In our simulations, we have never achieved the maximum memorization capacity of the model: increasing lateral connection strength, numerical issues arise, compromising the convergence of the model. Further investigations are needed to overcome these limitations.

In this work, we explored only a simple implementation of lateral connections, setting an equal strength between each pair of neurons. Several implementations are possible, providing different patterns of cortico-cortical connectivity matrix L, involving local or global interactions between neurons. The analysis of more complex competitiveness models will be investigated in future work.

## Data Availability

No new data were created or analyzed in this study. Data sharing is not applicable to this article.

## Appendix A

Both the MNIST and CIFAR-10 datasets were downloaded using the *scikit-learn* [36] Python package. The images were scaled in the range [0,1] to improve the learning efficiency of the model. For both the datasets, we trained a BCM network with 100 neurons, using ReLU activation function.

For the simulations on MNIST dataset, the network was trained for 500 epochs. Initial weights were sampled from a Gaussian distribution with mean 0 and standard deviation 0.1. Adam optimization algorithm was used, with batch size equal to 1000 and a constant learning rate of 0.04. The training was repeated for each lateral connections value in the set {−0.5, −0.05, −0.01, −0.005, 0, +0.002, +0.005, +0.006, +0.0065, +0.0068, +0.007, +0.0075, +0.01}.

For the simulations on the CIFAR dataset, the network was trained for 5000 epochs. Initial weights were sampled from a Gaussian distribution with mean 0 and standard deviation 0.02. Adam optimization algorithm was used, with batch size equal to 500 and a constant learning rate of 0.001. The training was repeated for each lateral connections value in the set {−0.5, −0.05, −0.01, −0.005, 0, +0.002, +0.005, +0.007, +0.01}.

The code developed for the reproducibility of the results is public available on Github [20].
